# CD44v6-Targeted Imaging of Head and Neck Squamous Cell Carcinoma: Antibody-Based Approaches

**DOI:** 10.1155/2017/2709547

**Published:** 2017-06-20

**Authors:** Diana Spiegelberg, Johan Nilvebrant

**Affiliations:** ^1^Department of Immunology, Genetics and Pathology, Uppsala University, Uppsala, Sweden; ^2^Division of Protein Technology, School of Biotechnology, Royal Institute of Technology, Stockholm, Sweden

## Abstract

Head and neck squamous cell carcinoma (HNSCC) is a common and severe cancer with low survival rate in advanced stages. Noninvasive imaging of prognostic and therapeutic biomarkers could provide valuable information for planning and monitoring of the different therapy options. Thus, there is a major interest in development of new tracers towards cancer-specific molecular targets to improve diagnostic imaging and treatment. CD44v6, an oncogenic variant of the cell surface molecule CD44, is a promising molecular target since it exhibits a unique expression pattern in HNSCC and is associated with drug- and radio-resistance. In this review we summarize results from preclinical and clinical investigations of radiolabeled anti-CD44v6 antibody-based tracers: full-length antibodies, Fab, F(ab′)_2_ fragments, and scFvs with particular focus on the engineering of various antibody formats and choice of radiolabel for the use as molecular imaging agents in HNSCC. We conclude that the current evidence points to CD44v6 imaging being a promising approach for providing more specific and sensitive diagnostic tools, leading to customized treatment decisions and functional diagnosis. Improved imaging tools hold promise to enable more effective treatment for head and neck cancer patients.

## 1. Introduction

### 1.1. Head and Neck Cancer

The term head and neck cancer summarizes malignancies of diverse origins, such as oral cavity, sinonasal cavity, salivary glands, pharynx, larynx, and lymph nodes in the head and neck. In spite of this diversity, the majority (about 95%) of head and neck cancers are squamous cell carcinomas (HNSCC) that arise from epithelial cells. HNSCC represents the sixth leading cause of cancer worldwide and results in approximately 0.5 million new diagnoses and approximately 0.3 million deaths annually [1]. Thus, HNSCC is a common cancer with low survival rate in advanced stages.

Important risk factors for head and neck cancers in Western countries include use of tobacco or alcohol and poor dietary intake. In many Asian countries, parts of East Africa, and the tropical Pacific, chewing areca or betel nuts and smoking bidis are contributing factors [2]. Moreover, exposure to Epstein-Barr virus (EBV) has been implicated in nasopharyngeal and hypopharyngeal carcinoma and infections with human papillomavirus (HPV), a causative agent of genital and anal cancers, can be a risk factor in developing oropharyngeal HNSCC [3, 4]. HPV-related HNSCCs are more frequent in young male adults and are generally associated with better outcome. In recent years, the incidence rate of HPV-associated HNSCC has increased rapidly while that of tobacco-associated HNSCC has declined, the latter of which is probably correlated to a general trend of fewer heavy smokers [3, 4].

The current multiple-modality treatment options with surgery, radiation, and chemotherapy are effective in early-stage disease and often curative. However, considering the delicate areas of face, head, and neck, treatment is associated with severe adverse outcomes, for example, on appearance and facial expression or on speech and swallowing function, which can substantially lower the quality of life. Moreover, a majority of HNSCC patients present with high-grade histology and with metastases located primarily in regional lymph nodes in the neck area. Despite recent advances in the use of chemotherapy with radiation and the use of hyperfractionated radiotherapy, advanced-stage HNSCC is still difficult to cure and the overall five-year survival rate is below 40–50% [4]. The low survival rate has been linked to high local recurrence rates, emergence of second primary disease, and development of distant metastases [9]. Earlier and more precise diagnosis could improve these numbers dramatically. Thus, there is a high demand for improved functional and molecular diagnostic tools such as radioimmunotargeting techniques against HNSCC-specific biomarkers. This review focuses on antibody-based imaging probes targeting CD44v6, a cancer-related cell surface variant of CD44, which exhibits unique expression patterns in HNSCC and is a promising target for radioimmunotargeting.

### 1.2. Radioimmunodiagnostics

Today, TNM staging of malignant tumors (TNM refers to size of primary tumor, number of regional lymph nodes, and distant metastases involved) is the fundamental basis for diagnosis, treatment planning, and recovery as well as posttreatment assessment. Physical and intraoperative examination, X-ray tomography, and pathological assessment are commonly employed for staging [10]. However, molecular and functional studies of biological processes in real time as well as biomarker visualization and evaluation may provide important information that is unattainable with traditional techniques. Noninvasive nuclear medical imaging, magnetic resonance imaging and spectroscopy (MRI and MRS, resp.), optical imaging by, for example, near-infrared fluorescence, and ultrasound might improve the accuracy of tumor detection. These procedures are useful for a wide range of applications including diagnostics, drug discovery and development, theranostics, and personalized medicine. Traditionally, evaluation of disease has to a large extent been based on anatomical data without connection to the underlying biology. For instance, changes in tumor size are used as an indicator for treatment response according to response evaluation criteria in solid tumors (RECIST) [11]. However, this can be misleading in many ways, for example, when the main bulk of the tumor consists of nontumorigenic cells that are more easily killed or in assessment of drugs that stabilize disease. Therefore, alternative indicators for treatment response are needed, such as the precise measurement of expression level of therapeutic targets or biomarkers. This type of detailed information on a per-patient basis is a prerequisite for effective targeted cancer therapy. Moreover, it enables monitoring of the treatment response of the targeted molecular therapy since it allows for repetitive noninvasive assessments. Here, molecular and functional imaging techniques have many advantages because they permit the investigation of the whole tumor burden in the body, thereby allowing assessment of biomarker expression and heterogeneity of the disease.

Radioimmunodiagnostic imaging techniques, including positron emission tomography (PET) and single-photon emission computer tomography (SPECT), are most useful in combination with computerized tomography (CT) or MRI scans, often referred to as multimodality imaging, which enable morphological evaluation and colocalization of the tracer at a precise anatomical position [14, 15]. Important properties of a selection of radionuclides that can be used for nuclear imaging and therapy are summarized in Table 1. SPECT imaging uses targeting vectors labeled with radionuclides that emit gamma ray photons or high-energy X-ray photons (e.g., ^99m^Tc, ^111^In, and ^177^Lu), with an energy range of 100–300 keV [16]. One photon is detected at a time by a single or a set of collimated radiation detectors. In PET imaging, radioisotopes that undergo positron emission decay can be used including ^11^C, ^18^F, ^64^Cu, ^68^Ga, ^89^Zr, and ^124^I [17–19]. Here, two oppositely directed (180°) 511 keV photons are emitted that can be registered by a circular scanner via coincident detection. By tracking the photons, computer simulations reconstruct 3D-images of the source of the annihilation. PET imaging has many advantages compared with SPECT, in particular a higher sensitivity and spatial resolution.

Today, ^18^F is the most commonly used isotope for PET imaging and ^18^F-fluorodeoxyglucose (^18^F-FDG) has become the golden standard PET-tracer in nuclear medicine and molecular imaging. It is used to measure increased glucose uptake (metabolism) and is measured as a standardized uptake value (SUV). A high SUV indicates an area of highly proliferating tissues. ^18^F-FDG PET/CT and PET/MRI are increasingly used in imaging of the head and neck area in order to add diagnostic information beyond pure anatomical data. Several clinical studies have compared the diagnostic performance of ^18^F-FDG PET/CT with that of PET/MRI since the superb soft-tissue resolution of MRI was expected to be of particular benefit for evaluating head and neck cancer [20]. However, the sensitivity of PET/CT in this application was comparable to that of PET/MRI. More recently, molecular and functional imaging techniques have improved dramatically, and several direct alternatives to imaging by ^18^F-FDG have been developed. One example is diffusion-weighted MRI, which can provide functional information based on direct measurement of the Brownian (random) motion of extracellular water molecules. This motion is restricted in hypercellular tumor tissue and quantified by a decrease in apparent diffusion coefficient. Changes in apparent diffusion coefficient values have also been linked with cell proliferation [10, 11] and to local tumor necrosis [21]. Another example is diffusion tensor imaging (DT MRI), which can be used to localize nerve bundles connected to malignant tissues and thereby potentially help guide surgery to better maintain the facial expression and communication abilities of the patient.

Although the diagnostic ability of PET/CT can be comparable to that of CT or MRI, depending on cancer type, ^18^F-FDG PET/CT can more effectively be used in staging of nodal disease and finding distant metastases or a second primary tumor. Such findings can significantly alter therapy decision-making. However, increased ^18^F-FDG uptake can also occur in nonmalignant areas due to posttreatment reactions, lymphadenitis, inflammation, and brown adipose tissue activation [22, 23]. Inflammation resulting from primary tumor ulceration or a recent biopsy can increase FDG uptake in lymph nodes and result in false-positive or equivocal activity. Similar problems occur at postsurgical sites, which are prone to inflammation especially after irradiation. Therefore it is important to find the right time frame for ^18^F-FDG PET after radiation treatment (about 8–12 weeks after radiotherapy) to reduce false-positive results associated with inflammation [23].

Taken together, these problems highlight the need of novel diagnostic methods with the high sensitivity of ^18^F-FDG-PET and increased tumor specificity. In HNSCC, targeted therapy or antibody-mediated diagnostic methods hold particular promise to improve early detection [24] and to treat minimal residual disease [25, 26].

### 1.3. Antibody-Based Molecular Imaging and Therapy 

Therapeutic antibodies have been approved for several cancers. Cetuximab (Erbitux®), which is a monoclonal antibody (mAb) targeting epidermal growth factor receptor 1 (EGFR), was approved by the US Food and Drug Administration (USFDA) for treatment of local or regionally advanced HNSCC in 2006. When used in combination with radiation therapy, it was shown to exhibit a survival benefit over radiation therapy alone. In 2011 cetuximab was also approved together with chemotherapy for recurrent or metastatic HNSCC. More recently (in 2016) pembrolizumab (Keytruda®), an immune checkpoint inhibitor, was granted accelerated approval for recurrent and metastatic HNSCC. Promising initial results have also been obtained using another checkpoint inhibitor, nivolumab (Opdivo®), which was approved to treat patients with head and neck cancer a few months later. Bevacizumab (Avastin®), an antibody that blocks angiogenesis by binding to vascular endothelial growth factor A (VEGF-A), is being evaluated for use in locally advanced HNSCC [27].

Furthermore, antibody-based molecular imaging or immuno-PET is a promising strategy [28, 29]. This approach allows the combination of high sensitivity and high resolution of, for example, a PET-scanner with the tumor specificity of a tumor targeting antibody. Further advantages of radioimmunotargeting include the capability for monitoring therapy response, dosimetric calculations, and therapy [30].

Full-length antibodies (~150 kDa) or smaller antibody derivatives are the most studied molecules for nuclear imaging and radioimmunotherapy, and there are several advantages to their use as radioimmunotargeting agents. The primary factors are economical and relatively simple production techniques together with high affinity. One early problem in this field was severe immune reactions from, for example, murine monoclonal antibodies, which has now been overcome by use of humanization techniques or de novo generation of human antibodies via, for example, in vitro selection. The comparatively large size of antibodies results in long duration in the circulation during the targeting phase of the tumor and a slow clearance from the bloodstream, which is beneficial for radioimmunotherapy. In contrast, these properties may be suboptimal for radioimmunodiagnostics where smaller molecules with fast biodistribution are generally preferred. When choosing radionuclide species to couple to the mAb, the choice is highly dependent on the antibody used, properties of its antigen, and what targeting concept is intended. The most important factors are decay half-life, availability, cost, and chemical nuclide properties for compatibility with the targeting vehicle (Table 1). Especially for targeted radioimmunotherapy, the radiation type, conjugate properties, and target tumor size must be taken into consideration. Generally, the most used radionuclides in targeted radioimmunotherapy are *β*-emitters, but *α*-emitters and Auger electron-emitting radionuclides can be used as well.

A recent review lists about 30 ongoing clinical trials evaluating the utility of antibody-based PET tracers using USFDA-approved and/or experimental antibodies in various cancer types, including glioblastoma, esophagogastric, breast, prostate, and colorectal cancer [28]. These probes target VEGF-A (bevacizumab), PGF (RO5323441), HER2 (trastuzumab), PSMA (Df-IAB2M, HuJ591), STEAP1 (MSTP2109A), MSLN (MMOT0530A), or EGFR (cetuximab) among others. Generally, cell surface receptors that are exclusively expressed by tumor cells are suitable targets for radioimmunodiagnostics. There are several promising receptors for radioimmunodiagnostics in head and neck cancer such as EGFR or isoforms of CD44. EGFR is one of the most ubiquitously overexpressed receptors with an increased expression level in more than 80% of cases. Molecular imaging using radiolabeled anti-EGFR antibody-based probes is therefore highly interesting and currently under clinical and preclinical investigation [31, 32]. However, clinical visualization of EGFR has not been very successful due to EGFR expression in nontumor tissues. For example ^99m^Tc-EC cetuximab (C225) or ^89^Zr-cetuximab showed a rather high uptake in liver as well as uneven distribution within the patient without an evident specific uptake of the tracer within the tumor [33, 34]. Although the incidence of distant metastases in HNSCC is relatively small in comparison to other cancer types, one of the major metastatic sites of HNSCC is the liver [35], which therefore complicates imaging with EGFR targeting probes.

Another promising target for radioimmunodiagnostics of HNSCC is CD44v6, an oncogenic splice variant of the cell surface receptor CD44. CD44v6 is currently the most established tumor antigen among the CD44 splice variants, with a large expression difference between healthy and malignant tissue, which is a key advantage for molecular imaging. In contrast to EGFR expression, CD44v6 expression in organs for distant metastases of HNSCC, such as the liver, is negligible.

### 1.4. CD44 and CD44v6

CD44 is one of the major receptors for the glycosaminoglycan hyaluronan, which is an abundant component of the extracellular matrix. However, CD44 also interacts with collagen, laminin, fibronectin, and cytokines and has been suggested to function as a coreceptor for numerous transmembrane proteins, for example, growth factor receptors [36, 37]. Additionally, CD44 expression has been linked to stem cell-like properties as well as tumor progression, cell migration, invasion, metastasis, and poor response to chemo- and radiotherapy [38–43].

**Figure 1:**
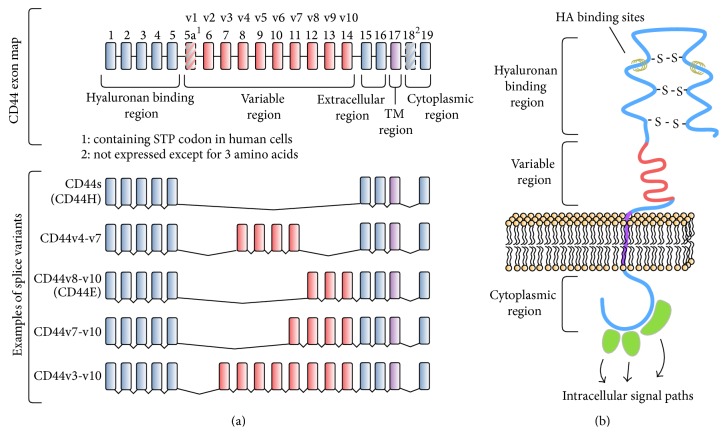
*CD44.* (a) Gene map of CD44. Standard CD44 (CD44s) does not contain variable exons. Exons v1–v10 are alternatively spliced. (b) Schematic overview of CD44. CD44 is a transmembrane protein, which consists of a cytoplasmic and extracellular region with hyaluronan binding sites and a variable region. HA: hyaluronic acid; TM: transmembrane region.

A single gene on chromosome 11p13 encodes CD44, which consists of 20 exons. Differential expression can give rise to a large number of CD44 isoforms. The standard form, referred to as CD44s (or CD44H, due to localization in hematopoietic cells), is the smallest and most abundant member of this large and heterogeneous family of multifunctional glycoproteins and is encoded by exons 1–5 and 15–20. The ten variably expressed exons that are lacking in CD44s are referred to as CD44v1-10 or exons 5a-15 in standard nomenclature [39] (Figures 1(a) and 1(b)). In humans, exon v1 contains a stop codon and no isoform containing this exon has been observed. Single exons or combinations of exon v2 to v10 can be inserted into the mRNA via alternative splicing translating into variations within the extracellular domain, which results in numerous protein variants. Furthermore, a multitude of posttranslational modifications, such as N- and O-glycosylation or palmitoylation, can further increase the diversity of CD44 gene products [44].

In humans, 19 different splice variants, the roles of which are not fully understood, have been identified at various expression levels in different tissues [45]. One example is CD44v7-v10 (CD44E), which is associated with normal epithelial cells. Several studies have associated certain CD44 splice variants with tumor cell invasion, metastasis, and disease progression, in particular isoforms containing CD44 exon variant 6 (CD44v6). Further studies have demonstrated high CD44v6 expression in several cancers, including breast, gastrointestinal, hepatocellular, and colorectal cancer and HNSCC [36, 42, 46, 47].

Overexpression of CD44v6 has been shown in squamous cell carcinomas, for example, in head and neck, lung, skin, esophagus, and cervix cancer [46]. However, CD44v6 expression frequencies vary throughout literature due to different detection methods (on RNA or protein level), different scoring systems, and the use of inapplicable antibodies [46]. However, overexpression of CD44v6 has been observed in over 90% of primary and metastatic HNSCC [44, 48]. Since CD44v6 is involved in progression of the disease and associated with radio-resistance, it is also an attractive therapeutic target [25]. Identifying differentially expressed diagnostic targets that are also involved in disease progression opens for theranostic applications, which combine diagnostic imaging with therapy by delivering therapeutic drugs and imaging vectors simultaneously [40]. Thus, monitoring of the disease can be followed by personalized treatment utilizing the same agent. Therapeutic radionuclides that can be used for molecular imaging, for example, ^177^Lu, are of particular interest in this approach [49, 50].

### 1.5. Antibody-Based Targeting of CD44v6 

Due to the high and homogenous expression of CD44v6 in HNSCC, antibodies recognizing this antigen have considerable potential for diagnosis and therapy [26]. In early studies, coinjection of a CD44v6-specific antibody together with metastatic cells was shown to retard or block metastatic spread in vivo [51, 52], which prompted the generation of antibodies specific for human CD44v6 [46]. U36 and BIWA 1 represent broadly used anti-CD44v6 mAbs and their encouraging targeting abilities have inspired more recent antibody engineering efforts.

**Figure 2:**
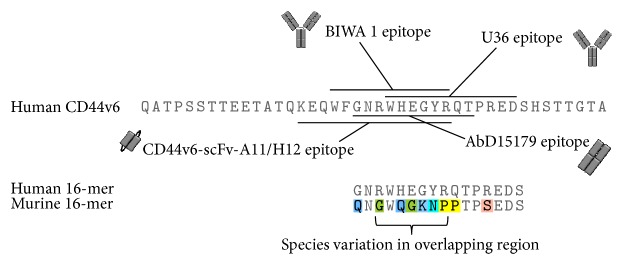
*Amino acid sequence of the human CD44v6 exon and antibody epitopes*. Antibodies U36 and BIWA 1 target overlapping epitopes in the v6 region [5, 6]. Recombinant Fab-fragment AbD15179 was generated using a peptide that overlaps with the epitopes of U36 and BIWA 1 [7]. CD44v6-specific scFvs recognize an epitope in the same region (Haylock et al. [8]). It is noticeable that murine CD44v6 has a low homology with the human sequence in the region where common antibody epitopes overlap, which is indicated in the alignment of a 14-residue region from the two species.

**Figure 3:**
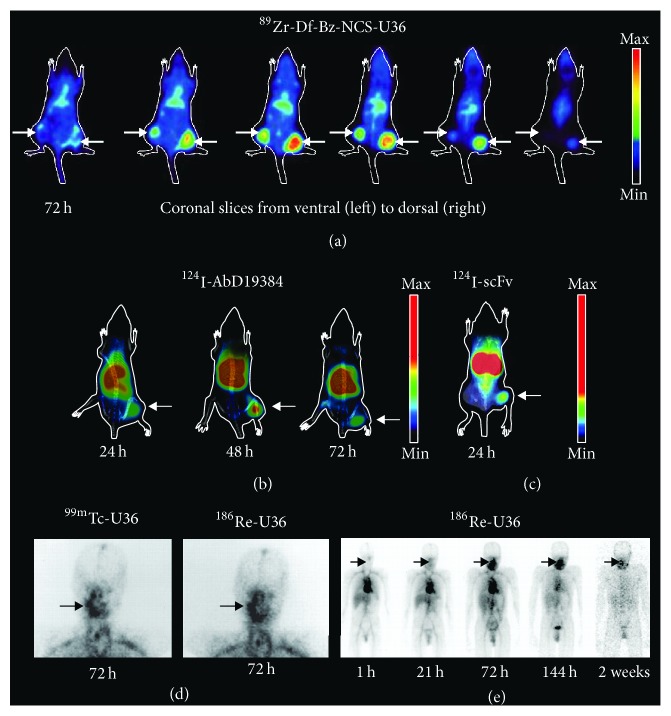
*Preclinical (a–c) and clinical (d–e) images of CD44v6 radionuclide targeting*. (a) Representative small animal PET images of a nude mouse bearing two head and neck cancer FaDu xenografts, obtained at 72 h after i.v. injection of the antibody conjugate ^89^Zr–Df–Bz–NCS–cmAb U36 (reprinted and modified from Vosjan et al.) [12]. (b) Representative small animal PET/CT images of nude mice bearing a squamous cell carcinoma A431 xenograft, obtained at 24 h, 48 h, and 72 h after i.v. injection of the human bivalent antibody fragment ^124^I-AbD19384. (c) Representative small animal PET/CT image of a nude mice bearing a squamous cell carcinoma A431 xenograft, obtained at 24 h after i.v. injection of an anti-CD44v6 targeting scFv fragment. (d) Planar imaging of head and neck region of a HNSCC patient 21 h after i.v. injection of the antibody conjugates ^99m^Tc-cmAb U36 (left) and ^186^Re-cmAb U36 (right) (reprinted and modified from Colnot et al. J. Nucl. Med. 2000) [13]. (e) Whole-body scans of a HNSCC patient 1 h, 21 h, 72 h, 144 h, and 2 weeks after i.v. injection of the antibody conjugate ^186^Re-cmAb U36 (reprinted and modified from Colnot et al. J. Nucl. Med. 2000) [13].

mAb U36 was selected from a panel of antibodies generated by immunization of mice with human HNSCC cells followed by generation of hybridoma clones [53]. Based on immunohistochemical staining of HNSCC tumors, U36 appeared to be the most promising antibody for targeting of CD44v6 with a stronger and more specific staining pattern relative to the best currently available mAb (E48) [54]. U36 recognizes a linear epitope in the v6 region of CD44v6 positive isoforms without cross-reactivity to murine CD44v6 [6, 53, 55], which has a low sequence homology in the targeted region (Figure 2). Radiolabeled U36 was shown to have high potential for in vivo targeting of HNSCC xenografts in mice as well as in human patients (Figure 3) [53, 56]. These promising data inspired a radioimmunotherapy (RIT) trial for the treatment of minimal residual disease in patients with head and neck cancer using ^186^Re-labeled chimeric (cmAb) U36 [13], in which the variable domains were transferred to a human IgG1 framework by previously developed strategies used for mAb E48 [57]. Radiolabeled cmAb U36 was well tolerated and displayed excellent targeting of tumor lesions. Moreover, stable disease and reduced tumor size were observed in some patients. However, the chimeric antibody still induced human antibody responses, which is an important consideration when repeated dosing is required for, for example, scouting studies prior to therapy.

Depending on the type of radionuclide chosen and the properties of the antibody-based targeting molecule, direct and indirect radiolabeling methods can be applied. Radioiodination with ^123^I, ^131^I, or ^124^I as well as, for example, radiolabeling of ^11^C compounds can be prepared by isotopic substitution, a direct exchange of stable atoms with radioisotopes of the same element. However, a majority of radiopharmaceuticals are prepared by introduction of a foreign element, as, for instance, for ^18^F-FDG where an ^18^F atom is introduced into the deoxyglucose molecule. Antibody-based targeting molecules can also be labeled with radiometals, for example, with ^99m^Tc or ^111^In, using the metal chelation method. For some probes a bifunctional chelate has to be introduced prior chelation of the radiometal. In this case, the radiometal is not directly incorporated into the molecule.

A large number of studies have demonstrated successful direct and indirect radiolabeling and use of mAb U36, its chimeric derivative, or smaller U36-derived antibody fragments in vitro and in vivo using, for example, ^88^Y [58], ^89^Zr [59], ^99m^Tc [56], ^111^In [60], ^124^I [61], ^125^I [62, 63], ^131^I [61], ^177^Lu [60], or ^211^At [64]. Thus, a diversity of labeling strategies and nuclides is available to fine-tune labeling, half-life, and dosimetry (e.g., estimation of radiation dose delivery to tumor and normal tissue) for applications in imaging or therapy. HNSCC is intrinsically radiosensitive, which may favor radioimmunotherapy. ^186^Re has been suggested to be better suited than ^131^I for RIT due to its lower gamma emission and higher conjugate stability. Labeling with ^186^Re using S-benzoyl mercaptoacetyltriglycide on lysine residues of the antibody [65] has been systematically evaluated. Adding too many payloads per antibody (>8) compromised immunoreactivity and resulted in faster clearance [66]. These results were later confirmed in a clinical study [67].

mAb BIWA 1, which was initially called VFF18, was generated by immunization of mice with recombinant CD44v3–v10 protein [5]. ELISA screening of hybridoma supernatants was used to identify CD44v6-specific mAbs and BIWA 1 was selected based on high affinity and specificity for human tumor cells in immunohistochemistry. Synthetic peptides were used to map the BIWA 1 epitope to a sequence that partially overlaps with the U36 epitope (Figure 2). In analogy with U36, binding was specific for human CD44v6 over its murine ortholog [5]. BIWA 1 was used for comprehensive immunohistochemical screening of tumor tissues, which demonstrated high and homogenous CD44v6 expression in a majority of analyzed tumors derived from squamous epithelium [5]. The same study demonstrated feasibility of targeting of CD44v6-expressing xenografts in mice using radiolabeled BIWA 1. Importantly, reactivity with normal human tissues was observed only on a subset of epithelial tissues but not on nonepithelial tissues [68]. As a first step towards human therapy, the safety, biodistribution, and tumor targeting potential of ^99m^Tc-labeled BIWA 1 were evaluated in HNSCC patients [69]. The results indicated that this antibody could be safely administered and achieve high and specific uptake in tumors, which enabled visualization already at time points before optimal tumor to nontumor ratios could be achieved. Similar tumor uptake at a higher and lower dose indicated that the high affinity of BIWA 1 might restrict tumor distribution as a result of saturation at a binding site barrier. In contrast, U36 displayed more homogenous distribution within the tumor at higher doses [56], which may be attributed to the ca. 35-fold lower affinity of U36 compared to BIWA 1 [48, 69].

### 1.6. Humanization and Evaluation of Drug Conjugated Antibodies 

Immunogenicity of the murine BIWA 1, which is linked to rapid clearance and allergic reactions, spurred the development of a humanized variant called BIWA 4 for further studies. Moreover, the high affinity mAb BIWA 1 showed complex formation with soluble CD44v6 in the blood and heterogeneous tumor uptake, which suggested that a lower affinity might be beneficial. Interestingly, a comparison of U36, BIWA 1, a chimeric antibody and two humanized variants of BIWA 1 designated BIWA 2, BIWA 4, and BIWA 8, respectively, revealed that lower affinity mAbs displayed superior tumor targeting capacities in mouse xenograft models [48]. Thus, the intermediate affinity, humanized mAb BIWA 4 (bivatuzumab), was selected for further clinical development over the higher affinity variant BIWA 8. In a following study on HNSCC patients, administration of ^99m^Tc-labeled BIWA 4 was well tolerated and no human anti-human antibody [35] responses were observed [70], which can be compared to a HAMA response in ca. 90% of patients treated with the parental murine BIWA 1. An intermediate dose level of 50 mg gave the highest tumor uptake and tumor to nontumor ratios. The lack of immunogenicity of BIWA 4 supported multiple administrations for radioimmunotherapy, which was evaluated in dose escalation studies using ^186^Re-labeled BIWA 4 on patients with advanced HNSCC [71, 72] as well as in patients with early-stage breast cancer [73]. Although radiolabeled BIWA 4 could be safely administered in all studies with tolerable side effects and only a few reported HAHA-responses, the results showed that uptake ratios were unfavorable in the breast cancer study. Thus, HNSCC remained the indication in focus for bivatuzumab due to a more favorable biodistribution likely resulting from higher and more specific expression of CD44v6 in HNSCC.

Although it was not the primary study objective, phase I RIT studies using ^186^Re-labeled cmAb U36 or BIWA 4 showed promising antitumor effects with consistent stable disease at higher dose levels [74]. At the time the first antibody-drug conjugate (ADC), gemtuzumab ozogamicin (Mylotarg®) that targets CD33, had already been approved for the treatment of amyloid myeloid leukemia. An ADC combines the targeting capability of an antibody with a cytotoxic payload with cancer-killing ability. Hence, it was envisioned that coupling of BIWA 4 to a cytotoxic drug instead of a radionuclide might provide a more effective immunoconjugate for adjuvant therapy of HNSCC. Mertansine (also called DM1) is a derivative of the antimicrotubule agent maytansine with more than 100-fold higher cytotoxic activity compared to other clinically used anticancer drugs such as anthracyclines or taxanes [75]. The antibody-drug conjugate was designed to release and activate the cytotoxic, disulfide-linked, part upon cellular internalization. Initial preclinical evaluation in animals demonstrated dose-dependent efficacy with long-lasting tumor regression of mertansine conjugated to bivatuzumab (BIWI 1 or bivatuzumab mertansine) whereas no effects were seen on tumor growth for the unconjugated antibody [74, 76]. However, in spite of promising results in several studies, death of one patient from drug related toxic epidermal necrolysis during a phase I dose-escalating study led to premature termination of the study [74, 76]. Arguably, expression of CD44v6 is not sufficiently selective for tumor cells to allow systematic administration of antibody conjugates containing highly toxic agents like mertansine or the linker was not sufficiently stable to prevent exposure to nontumor tissue [76]. Interestingly, bivatuzumab mertansine improved local tumor control with acceptable systemic toxicity in a murine model when administered at a lower dose in combination with fractionated irradiation [77]. Furthermore, one of only two currently approved ADCs, for example, trastuzumab emtansine (Kadcyla®), utilizes the same toxin with a noncleavable linker. It is also noteworthy that gemtuzumab ozogamicin, the first ADC to be approved, was withdrawn from market in 2010 when a large study failed to demonstrate that it extended survival over conventional therapy and was associated with a high rate of fatal toxicity (USFDA). Taken together, this illustrates that while the concept of ADCs is relatively straightforward, the design of a functional and effective antibody-drug conjugate is very challenging.

In spite of advances in therapeutic intervention, the early detection of cancers is still important to improve the clinical outcome for cancer patients. Diagnostic use of radiolabeled antibodies can tolerate expression of the target antigen in normal tissues, especially in an area outside of the anatomical region of interest or in normal tissue that is poorly accessible to antibodies [25]. Several radioimmunoconjugates have been approved for cancer diagnosis, for example, arcitumomab (CEA-scan®), a ^99m^Tc-labeled antibody fragment used for imaging of colorectal cancer, and capromab pendetide (ProstaScint®), an ^111^In-labeled mAb directed against prostate specific membrane antigen (PSMA) [78]. In the case of CD44v6-targeting in HNSCC, selection of an appropriate radioimmunoconjugate may also help overcoming treatment-related skin toxicity [26]. The promises of radioimmunodiagnostics and recent advances in antibody engineering have inspired the development of a new generation of antibodies targeting CD44v6.

### 1.7. Recombinant Antibodies and Antibody Engineering 

**Figure 4:**
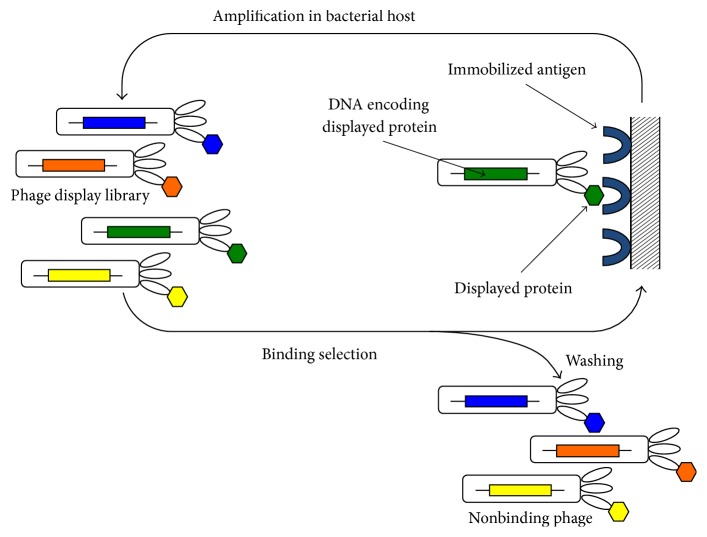
*Phage display selection from a protein library*. Protein libraries are displayed on phage particles as fusions to coat proteins. Each phage displays a unique protein and encapsulates the encoding DNA, which links the genotype and phenotype of the displayed protein. Protein variants (e.g., antibodies) that bind an immobilized antigen are isolated through rounds of binding selection and amplification. Nonbinding phages are removed by washing. Retained phages are recovered, amplified by bacterial infection, and cycled through additional rounds of selection. Compared to immunization-based methods, in vitro selection enables full control of library design and selection conditions. Binding clones are identified through sequencing of the encapsulated DNA.

The high immunogenicity and weak interaction with human complement and Fc*γ* receptors of murine antibodies generally translate into a low success rate in medical development [79]. Using recombinant DNA technology, chimeric antibodies, which consist of human constant domains with murine variable regions (e.g., rituximab, Rituxan®; 2006) and humanized antibodies, where mainly the complementarity determining regions (CDRs) are of nonhuman origin (e.g., daclizumab, Zinbryta®; 2003), can be generated. However, these hybrid antibodies still carry foreign sequence in their antigen-binding loops, which may lead to immunogenicity as exemplified by the HAHA-responses observed in two patients in a phase I therapy study using ^186^Re-labeled BIWA 4 [71]. Moreover, humanized antibodies frequently loose binding affinity in the process of loop grafting or framework engineering. Human mAbs are defined as having variable domains that are entirely derived from human antibody repertoires. Adalimumab (Humira®) was the first fully human antibody to be approved for human therapy in 2002. It was generated by in vitro display without animal immunization or hybridoma technology. Display methods physically link an antibody fragment to its encoding DNA and thereby enable screening of libraries containing billions of variants in vitro (Figure 4). Rounds of selection and amplification are employed to enrich antigen-binding clones with desired properties. The sequences of promising variants are immediately available, which facilitates further engineering of antibody properties including affinity, valency, and stability. In vitro selection has several advantages over traditional immunization-based antibody generation. It enables full control over the selection conditions and the epitopes that are targeted. For example, alternating selection on orthologs of relevance for future testing in animal models can be applied to isolate clones that display cross-species binding. In contrast, since antibodies that are reactive to self are eliminated, it is almost impossible to raise antibodies against epitopes that are highly conserved across species using immunization. Today, most antibodies that enter clinical trials are completely human and are derived from phage display technology or transgenic mice, which have been engineered to carry human antibody repertoires [80–82].

Antibody fragments are commonly used in the engineering of antibody properties and have an increasing clinical importance [83]. The fragment antigen-binding (Fab) is a heterodimer consisting of the light chain and the variable and first constant domains of the heavy chain. A single chain fragment variable (scFv) consists of the light and heavy variable domains connected by a linker. Fabs are generally more stable than scFvs and activity is better retained upon conversion to full-length IgG. Single domain formats derived from variable [84, 85] or constant domains [86, 87] represent the smallest human antibody fragments. Building on the modular architecture of antibodies, many innovative formats with diverse valences and antigen-binding specificities have been constructed [88].

Using phage display we have isolated CD44v6-binding fully human Fab fragments that bind a defined peptide that overlaps with the epitopes of U36 and BIWA 1 [7] (Figure 2). Fab AbD15179 was selected as a lead candidate among eight clones derived from the HuCAL Platinum synthetic antibody library [89]. All selected antibodies displayed competition with U36, which indicated that the epitope-guided selection was successful. AbD15179 specifically recognized a CD44v6-positive isoform with low nanomolar affinity without measurable cross-reactivity to CD44v6-negative controls [7]. The in vivo targeting properties of radiolabeled AbD15179 were evaluated in tumor-bearing mice [90]. In general, Fab fragments exhibit shorter half-life, faster blood clearance, and better tumor to background ratios compared to full-length antibodies and are thus promising for tumor imaging applications. The human Fab had a favorable biodistribution and could discriminate between high and low CD44v6 expressing tumors in vivo. Notably, the labeling approach can influence the kinetic properties of the antibody conjugate. This was demonstrated for AbD15179 using different squamous cell carcinoma cell lines [91] and highlights the importance of functional assessment of the radioimmunoconjugate. Reformatting AbD15179 into a bivalent construct followed by radiolabeling resulted in a tracer (^124^I-AbD19384) with slower target dissociation that displayed more favorable tumor imaging properties when compared to ^18^F-FDG PET (Figure 3) [92]. Similar results speaking in favor of a smaller bivalent antibody fragment were obtained when a Fab, a bivalent F(ab′)_2_, and a full-length version of mAb U36 were compared side by side in vitro and in mice carrying CD44v6-expressing xenografts [63].

More recently we have generated a panel of scFvs that target an epitope that overlaps with Fab AbD15179 (Haylock et al. [8], Figure 2). CD44v6-specific scFvs were selected by phage display with negative selection on a CD44v6-negative isoform of CD44, which represented more ubiquitously expressed CD44 isoforms. V_H_ domains from CD44v6-specific first generation clones were next combined with a naïve V_L_ repertoire followed by stringent selection of high affinity clones. Two top candidates denoted CD44v6-scFv-A11 and CD44v6-scFv-H12 demonstrated specific binding to CD44v6-expressing cells in vitro with subnanomolar affinity. Both variants were radiolabeled using ^111^In or ^125^I and their tumor targeting abilities evaluated in tumor-bearing mice. Radiolabeled scFvs, in particular ^125^I-labeled fragments, provided high tumor-to-blood ratios and kinetics suitable for molecular imaging. Compared to, for example, Fab fragments, smaller antibody fragments like scFvs are expected to provide better imaging contrast as a result of a faster biodistribution and enhanced tissue penetration [78]. Despite the improved penetration of smaller fragments, the total tumor uptake is generally lower compared to full-length antibodies due to the shorter time in circulation. However, faster clearance and shorter circulation times are beneficial for tumor to organ ratio and contrast in molecular imaging. The smaller size of scFvs versus, for example, F(ab′)_2_ combined with high affinity monovalent binding yielded advantageous tumor to organ ratios already at 24 h p.i. (Haylock et al. [8]), which is half the time required to reach similar ratios for the F(ab′)_2_ fragment [92]. For imaging, the contrast between tumor and surrounding tissue is more important than the total tumor uptake and a high affinity is generally advantageous for radioimmunodiagnostic applications [25].

Several recent studies have reported on CD44v6-targeting antibody reagents. For example, human scFvs recognizing CD44v6 were isolated by phage display from a synthetic antibody library [93]. Using a similar strategy, Chen et al. selected CD44v6-binding scFvs from a library constructed from lymphocytes from human blood donors [94]. Interestingly, the single CD44v6-binding clone that was identified in this study was lacking the variable light domain. Thus, it will need more characterization of its biophysical properties and binding characteristics before it can be employed for, for example, tumor imaging in vivo. By immunizing mice with a 43-amino-acid region derived from v6 conjugated to a carrier protein, murine antibodies have also been generated [95]. However, in contrast to the antibodies generated in vitro by phage display, the sequences of these CD44v6 binders are unknown. Compared to other available CD44v6-binding recombinant antibodies, our Fab and scFv clones are more thoroughly characterized and have a demonstrated potential for tumor detection in vivo.

Antibodies targeting a v6-epitope have been shown to possess antitumor effects in vitro and in vivo [96–99], which imply that binding per se may promote a desirable phenotype. Intriguingly, these effects have been mapped to a three-residue peptide (RWH) [100] that is localized in the center of the mapped BIWA 1 epitope and also present in the epitopes recognized by our lead Fab and scFv clones (Figure 2).

## 2. Conclusion 

CD44v6-positive isoforms have been related to aggressive tumor behavior and are abundantly expressed particularly in squamous cell carcinoma of the head and neck. In spite of improvements in locoregional treatment, the rate of recurrence is still close to 40%, whereas ca. 25% of these patients also develop distant metastases [74]. Autopsy studies have shown incidences of distant metastases in up to 57% of cases [25]. Thus, there is a demand for new tools for early-stage diagnosis to improve patient outcomes. In addition, advanced-stage HNSCC patients frequently harbor residual tumor cells after surgery and radiotherapy. The role of adjuvant chemotherapy for this group of patients is limited, and therefore the development of an effective adjuvant systemic treatment targeting distant micrometastases and minimal residual disease is another major challenge.

CD44v6-targeted antibody-mediated diagnosis and therapy hold promise to provide more tumor specific alternatives. Several antibodies have shown promise in CD44v6-targeting and promoted the development of bivatuzumab mertansine, an antibody-drug conjugate designed to kill CD44v6-expressing tumor cells. Although effective, a low antigen expression in normal epithelial cells combined with a highly toxic payload resulted in skin toxicity and termination of the development program. Nonetheless, the combination of high sensitivity and resolution of PET with the specificity and affinity of an anti-CD44v6 mAb makes immuno-PET an attractive tumor detection modality. To achieve optimal tumor to nontumor ratio, a labeling method and radionuclide with suitable half-life for adequate tumor accumulation and nonspecific clearance has to be selected. Combined with advances in antibody engineering that enable easier optimization of antibody format and targeting properties, this offers a promising approach to develop novel immunoconjugates. For example, affinity and specificity can be fine-tuned in vitro and antibodies can be engineered to tolerate labeling with minimal functional interference. Fully human antibodies, which are expected to be less immunogenic and better tolerated in repeated dosing, can be engineered without a need for unpredictable immunization-based methods.

Capitalizing on these technological developments, we have established a new generation of fully human antibody fragments against CD44v6 with promising tumor targeting properties in vivo. Several questions remain to be answered before these reagents can be employed for use in humans. For example, the lack of cross-reactivity with murine CD44v6 makes the transferability of preclinical findings in mouse models difficult to predict. Preclinical studies using monkeys, which have a higher sequence homology in the targeted region [68], may provide a more suitable animal model. Moreover, potential immunogenicity, particularly upon repeated administration, has to be evaluated more thoroughly.

## Figures and Tables

**Table 1:** *Properties of selected diagnostic and therapeutic radionuclides*. Some low abundance emissions have been omitted for clarity. *β*+: positron emission, *γ*: gamma ray emission, PET: positron emission tomography, and SPECT: single-photon emission computed tomography.

Radionuclide	Half-life	Decay mode	Energy (keV)	Max range in tissue	Major application
^18^F	1.83 h	*β*+ (97%)	633		PET
^11^C	20.4 min	*β*+ (99.8%)	960		PET
^68^Ga	1.1 h	*β*+ (88%)	1899		PET
^89^Zr	3.3 d	*β*+ (23%)	897		PET
^64^Cu	12.7 h	*β*+ (17.8%)	653		PET
^124^I	4.17 d	*β*+ (25%)	2138, 1534		PET
^111^In	2.8 d	*γ* (90.6%), *γ* (94.1%)	171, 245		SPECT
^99m^Tc	6.0 h	*γ* (89%)	140.5		SPECT
^211^At	7.2 d	*α* (42%), *γ* (21%)	5868	0.8 mm	SPECT, radionuclide therapy
^177^Lu	6.7 d	*β*− (78.6%), *γ* (11%), *γ* (6%)	498, 208, 113	1.6 mm	SPECT, radionuclide therapy
^131^I	8.0 d	*β*− (89.9%), *γ* (81.7%)	606, 364	4 mm	SPECT, radionuclide therapy
^225^Ac	9.9 d	*α* (50.7%)	5830	0.9 mm	Radionuclide therapy
^90^Y	2.7 d	*β*− (99.9%)	2280	11 mm	Radionuclide therapy
^89^Sr	50.5 d	*β*− (100%)	1491	7 mm	Radionuclide therapy
^153^Sm	1.9 d	*β*− (44%), *β*− (34%)	702, 632	3.3 mm	Radionuclide therapy
